# Establish an allele-specific real-time PCR for *Leishmania* species identification

**DOI:** 10.1186/s40249-022-00992-y

**Published:** 2022-06-02

**Authors:** Yun Wu, Mengyuan Jiang, Shaogang Li, Nicholas R. Waterfield, Guowei Yang

**Affiliations:** 1 Beijing Institute of Tropical Medicine, Beijing Friendship Hospital, Capital Medical University, 95 Yong’an Road, Xi Cheng District, Beijing, 100050 China; 2 Warwick Medical School, Warwick University, Coventry, UK

**Keywords:** *Leishmania*, Species identification, Allele-specific real-time PCR, SNPs

## Abstract

**Background:**

Leishmaniasis is a serious neglected tropical disease that may lead to life-threatening outcome, which species are closely related to clinical diagnosis and patient management. The current *Leishmania* species determination method is not appropriate for clinical application. New *Leishmania* species identification tool is needed using clinical samples directly without isolation and cultivation of parasites.

**Methods:**

A probe-based allele-specific real-time PCR assay was established for *Leishmania* species identification between *Leishmania donovani* and *L. infantum* for visceral leishmaniasis (VL) and among *L. major*, *L. tropica* and *L. donovani/L. infantum* for cutaneous leishmaniasis (CL), targeting hypoxanthine-guanine phosphoribosyl transferase (HGPRT) and spermidine synthase (SPDSYN) gene with their species-specific single nucleotide polymorphisms (SNPs). The limit of detection of this assay was evaluated based on 8 repeated tests with intra-assay standard deviation < 0.5 and inter-assay coefficients of variability < 5%. The specificity of this assay was tested with DNA samples obtained from *Plasmodium falciparum*, *Toxoplasma gondii*, *Brucella melitensis* and *Orientia tsutsugamushi*. Total 42 clinical specimens were used to evaluate the ability of this assay for *Leishmania* species identification. The phylogenetic tree was constructed using HGPRT and SPDSYN gene fragments to validate the performance of this assay.

**Results:**

This new method was able to detect 3 and 12 parasites/reaction for VL and CL respectively, and exhibited no cross-reaction with *P. falciparum*, *T. gondii*, *B. melitensis*, *O. tsutsugamushi* and non-target species of *Leishmania.* Twenty-two samples from VL patients were identified as *L. donovani* (*n* = 3) and *L. infantum* (*n* = 19), and 20 specimens from CL patients were identified as *L. major* (*n* = 20), providing an agreement of 100% compared with sequencing results. For further validation, 29 sequences of HGPRT fragment from nine *Leishmania* species and 22 sequences from VL patients were used for phylogenetic analysis, which agreed with the results of this new method. Similar results were obtained with 43 sequences of SPDSYN fragment from 18 *Leishmania* species and 20 sequences from CL patients.

**Conclusions:**

Our assay provides a rapid and accurate tool for *Leishmania* species identification which is applicable for species-adapted therapeutic schedule and patient management.

**Graphical Abstract:**

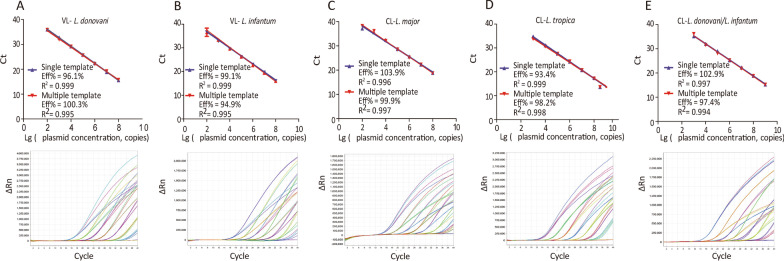

**Supplementary Information:**

The online version contains supplementary material available at 10.1186/s40249-022-00992-y.

## Background

Leishmaniasis is a zoonotic disease caused by as many as 21 species of *Leishmania*, which can lead to lethal or traumatic outcome and associated social stigmatization [1]. The vectors and animal hosts of *Leishmania* present diversity and intersectionality, making the diseases more complicated to control. Due to the infection with different species of *Leishmania*, many subclinical infections have no symptoms and many patients exhibit various clinical manifestations [2, 3]. Typically, visceral leishmaniasis (VL) is caused by *L. donovani* and *L. infantum*, which is a serious infection with internal organs and bone marrow and will have fatal consequence without treatment in time [4]. Cutaneous leishmaniasis (CL) and mucosal leishmaniasis (ML) are limited to the skin and mucous membranes, and caused by different *Leishmania* species. CL is caused by *L. major*, *L. tropica* and *L. infantum* which are prevalent around the Mediterranean basin, the Middle East, the horn of Africa, and the Indian subcontinent, and *L. amazonensis*, *L. chagasi* (sometimes still referred to as *L. infantum* in South America), *L. mexicana*, *L. naiffi*, *L. braziliensis* and *L. guyanensis* which are prevalent around Middle and South America [5]. *L. braziliensis* and *L. aethiopica* can cause overt ML [6]. Cured VL, infected with *L. infantum* and *L. donovani*, sometimes occurs post kala-azar dermal leishmaniasis (PKDL) [7, 8].

As different *Leishmania* species exhibit various virulence level, genetic heterogeneity and responses to chemical drugs, the outcome tended to be better when therapy was species-directed performed [9–11]. For instance, *L. major*, *L. donovani*, *L. braziliensis* (in Guatemala) and *L. tropica* are more sensitive to antimony compared to *L. aethiopica*, *L. panamensis* and *L. braziliensis* (in Brazil). Miltefosine is an effective drug for treating CL caused by *L. guyanensis*, *L. panamensis* and *L. donovani*, whereas CL caused by *L. infantum* and *L. braziliensis* exhibit more resistant to it. Unlike *L. tropica*, *L. major*, *L. mexicana* and *L. braziliensis* are more susceptible for paromomycin (PM). Amphotericin B is recommended to treat CL caused by *L. tropica*, *L. braziliensis*, *L. major* and *L. aethiopica* but not for *L. infantum* [12–16]. Further, as there are co-infections with different *Leishmania* species, it will lead to different pathogenicity and drug sensitivity which make the treatment more complicated [17, 18]. Thus, *Leishmania* species identification is important in treatment and patient management, including pharmaceutical selection, appropriate treatment determination (intralesional, intramuscular, oral systemic, or parenteral) and monitoring potential infection sequelae [19–22].

Traditional *Leishmania* diagnostic techniques, such as microscopic examination, protozoan culture in vitro and serological immunoassay, cannot identify *Leishmania* species. In present clinical practice, it is still based on empirical judgment according to the information of local epidemiology. However, it could make inappropriate determination for traveler and co-infections with different species [1]. There are some techniques were developed to discriminate *Leishmania* species, such as sequencing of individual gene, restriction fragment length polymorphism (RFLP), high resolution melting, multilocus sequencing typing and mass spectrometry [21, 23–30]. As the World Health Organization recommended, the “gold standard” method used to identify *Leishmania* species is multi-site enzyme electrophoresis (MLEE), which requires culture of parasites [2]. However, some *Leishmania* species are difficult to culture in vitro with cumbersome experiment procedure which also makes the results among different laboratories incomparable. Although some probe based real-time PCR assays were developed for *Leishmania* species identification, they are mainly focused on *L. mexicana*, *L. braziliensis*, *L. peruviania* and *L. major* for CL and not suitable for other common clinical infection related species [31, 32]. Thus, for clinical applications, a tool for *Leishmania* species identification among common clinical pathogens, such as *L. donovani, L. infantum, L. major *and* L. tropica*, is needed to be developed using clinical samples directly without isolation and cultivation of parasites.

In this study, to identify *Leishmania* species, hypoxanthine-guanine phosphoribosyl transferase (HGPRT) and spermidine synthase (SPDSYN) genes were selected from 34 housekeeping genes. Our results showed that, HGPRT gene with species-specific single nucleotide polymorphisms (SNPs) can identify parasite species between *L. donovani* and *L. infantum* for VL, and SPDSYN gene with species-specific SNPs can distinguish parasite species among *L. major*, *L. tropica* and *L. donovani*/*L. infantum* for CL. Thus, an allele-specific real-time PCR technique was established for *Leishmania* species identification with clinical specimens from VL and CL patients.

## Material and methods

### Patients and samples

A total of 42 clinical samples from patients at Beijing Friendship Hospital, Capital Medical University from July 2015 to Sep 2021 (Table 1). The bone marrow (*n* = 22) and skin lesion tissue (*n* = 20) were collected from patients with VL and CL individually for allele-specific real-time PCR testing. VL patients presented with symptoms such as fever, splenomegaly and/or hepatomegaly, *Leishmania* amastigotes found in their bone marrow samples under microscope or PCR positive or *Leishmania* parasite culture positive. CL patients appeared as ulcer and nodule/plaques features in which *Leishmania* amastigotes were identified under microscopy. All bone marrow and skin lesion tissue were stored at liquid nitrogen till use. DNA samples of *Plasmodium falciparum*, *Toxoplasma gondii*, *Brucella melitensis* and *Orientia tsutsugamushi* were used as non-leishmaniasis controls.

**Table 1:** Patients’ characteristics of Visceral *Leishmaniasis* and Cutaneous *Leishmaniasis*

Diseases	Patient ID	Age	Gender	Diagnosis basis				Laboratory test					Imaging	Combined infections	Parasite load	Treatment outcome	Combined HPS
				Region	Symptoms	Anti-rk39	Etiology	WBC	RBC	PLT	ALB	GLB					
VL	1	50	M	Yangquan, Shanxi, China	Fever, cough, diarrhea, abdominal pain, ascites	+	Culture	3.4	3.6	11	31.7	44.9	Enlarged spleen	HBV, Mycoplasma	1.9 × 10^5^	Cure	No
	2	51	M	Yangquan, Shanxi, China	Nausea and vomit, appetite, oily, weight loss	+	Microscopy	2.3	3.7	125	25.6	59	Enlarged liver and spleen	Herpes simplex virus, EB virus	1.8 × 10^5^	Under treatment	No
	3	1	F	Yangquan, Shanxi, China	Fever, listlessness	+	Microscopy	2.6	2.7	81	30.7	34.1	Enlarged liver and spleen	Bacterial pneumonia	1.2 × 10^7^	Under treatment	No
	4	2	M	Yangquan, Shanxi, China	Fever	+	PCR [54]	4.7	4.6	90	34.6	48.7	Enlarged liver and spleen	Mycoplasma, Rickettsia Q fever	un	Under treatment	No
	5	35	F	Linfen, Shanxi, China	Fever, chills	+	Microscopy	0.6	2.7	39	27.2	24.8	Enlarged spleen	Mycoplasma	2.1 × 10^5^	Cure	Yes
	6	66	M	Pingding, Shanxi, China	Fever, cough, fatigue	+	Microscopy	1.6	3.2	87	24.2	58.9	Enlarged spleen	None	8.7 × 10^5^	Cure	No
	7	53	M	Yangquan, Shanxi, China	Fever, night sweats, chills	+	Microscopy	3.6	3.2	156	23.3	63.1	Enlarged liver, Splenectomy	Fungal and bacterial pneumonia	4.2 × 10^6^	Cure	No
	8	44	M	Pingding, Shanxi, China	Fever, shortness of breath, fatigue, profuse sweating	−	Microscopy	1.7	2.3	44	29.7	20.4	Enlarged spleen	Candida albicans	un	Cure	Yes
	9	1	F	Xingtai, Hebei, China	Fever	+	Microscopy	11.8	4.2	281	38.8	42.7	Enlarged spleen	Neisseria, Mycoplasma	4.2 × 10^6^	Cure	Yes
	10	33	M	Weinan, Shaanxi, China	Fever, cough, expectoration	−	Microscopy	13	4.5	68	22.2	21.3	Enlarged spleen	Fungal and bacterial pneumonia, CytoMegalo virus	1.5 × 10^7^	Death	Yes
	11	42	M	Bayannaoer, Inner Mongolia, China	Fever, chills	+	Microscopy	2.06	3.18	453	31.7	28.5	Enlarged spleen	Epstein–Barr virus	1.0 × 10^8^	Under treatment	Yes
	12	26	M	Yangquan, Shanxi, China	Fever, chills	+	Microscopy	2.5	2.4	51	27.6	41	Enlarged spleen	None	1.7 × 10^7^	Cure	Yes
	13	26	F	Longnan, Gansu, China	Fever, chills	+	Microscopy	4.5	2.8	124	32.6	30.8	Enlarged liver and spleen	None	1.4 × 10^5^	Cure	Yes
	14	66	F	Yangquan, Shanxi, China	Chest tightness, fatigue, cough	+	Microscopy	2.4	2.8	64	25.6	74.7	Enlarged liver and spleen	Mycoplasma	6.3 × 10^6^	Cure	No
	15	32	M	Yangquan, Shanxi, China	Fever, chills, fatigue, headache, sweat profusely, cough	+	Microscopy	1.7	3.0	91	35.9	81.1	Enlarged spleen	None	None	Under treatment	No
	16	47	F	Yangquan, Shanxi, China	Fatigue, fever, chills, nausea and vomit	+	Microscopy	1	2.7	105	22.4	83.4	Enlarged spleen	Mycoplasma, Epstein-Barr virus, Sarkozy virus	1.7 × 10^5^	Under treatment	No
	17	3	M	Yangquan, Shanxi, China	Fever, listlessness, expectoration, vomit, abdominal pain, diarrhea	+	Microscopy	3.9	4.2	70	33.4	44	Enlarged spleen	None	7.9 × 10^5^	Under treatment	No
	18	30	M	Yangquan, Shanxi, China	Fever,	+	Culture	7.4	3.4	268	20.3	135	Enlarged liver, Splenectomy	Bacterial pneumonia	3.0 × 10^6^	Cure	No
	19	28	F	Gansu, China	No obvious symptoms	+	PCR [54]	3.5	4.2	125	33.5	26.2	Enlarged spleen	Mycoplasma	un	Under treatment	No
	20	80	M	Shanxi, China	Cough, fatigue	+	Microscopy	2.4	4.3	134	27.4	66.6	Enlarged spleen	Sarkozy virus, Adenovirus, Chlamydia	1.5 × 10^6^	Cure	No
	21	61	M	Beijing, China	Fever	+	Microscopy	5.8	4.7	148	34.4	38.3	Enlarged spleen	None	2.9 × 10^3^	Under treatment	Yes
	22	52	F	Shanxi, China	No obvious symptoms	−	Microscopy	1.5	4.6	191	42.8	26.8	Enlarged spleen	None	3.9 × 10^4^	Under treatment	No
CL	23	42	M	Iraq	Multiple skin ulcers	−	Microscopy	6	5	280	41.6	31.7	Normal	Mycoplasma, Staphylococcus aureus	1.7 × 10^7^	Cure	No
	24	47	M	Iraq	Multiple skin ulcers	−	Culture	5.5	4.5	182	35.8	31.1	Normal	unclear upper respiratory tract infection	1.5 × 10^8^	Cure	
	25	35	M	Iraq	Multiple skin ulcers	−	Culture	5.6	4.6	232	41.4	28	Normal	None	5.4 × 10^7^	Cure	
	26	55	F	Morocco	Multiple skin ulcers	+	Microscopy	5.2	4.4	196	38.4	31.8	Normal	None	1.0 × 10^8^	Cure	
	27	48	M	Iraq	Multiple skin ulcers	−	Microscopy	3.8	4.1	164	35.9	23.4	Normal	None	4.9 × 10^7^	Cure	
	28	29	M	Iraq	Multiple skin ulcers	−	Microscopy	4.9	5.2	264	40.8	22.7	Normal	Mycoplasma	1.4 × 10^6^	Cure	
	29	40	M	Iraq	Multiple skin ulcers	+	Microscopy	7.7	4.94	202	40.2	26.4	Normal	Chlamydia	8.0 × 10^4^	Cure	
	30	34	M	Iraq	Multiple skin ulcers	−	Microscopy	3.9	4.41	161	38.3	24.2	Normal	Mycoplasma	1.8 × 10^8^	Cure	
	31	43	M	Iraq	Multiple skin ulcers	−	Microscopy	4.5	4.6	207	39.5	29.9	Normal	Mycoplasma, Adenovirus	9.9 × 10^7^	Cure	
	32	40	M	Iraq	Multiple skin ulcers	−	Microscopy	5.1	5.0	162	43.8	26.5	Normal	EB virus	5.0 × 10^6^	Cure	
	33	31	M	Iraq	Multiple skin ulcers	−	Microscopy	8.4	5.5	57	48.5	24	Normal	None	3.2 × 10^7^	Cure	
	34	34	M	Iraq	Multiple skin ulcers	−	Microscopy	5.2	4.6	189	39	29.7	Enlarged spleen	Mycoplasma, Legionella	5.4 × 10^7^	Cure	
	35	42	M	Iraq	Multiple skin ulcers	−	Microscopy	5.2	4.6	203	41.7	29.2	Normal	Sarkozy virus, adenovirus, Mycoplasma	2.2 × 10^7^	Cure	
	36	40	M	Iraq	Multiple skin ulcers	−	Microscopy	6	4.9	243	42.3	26.2	Normal	None	2.0 × 10^8^	Cure	
	37	33	M	Iraq	Multiple skin ulcers	−	Microscopy	8.4	5.3	227	42.6	26.5	Normal	Mycoplasma, Chlamydia	9.1 × 10^7^	Cure	
	38	26	M	Nigeria	Multiple skin ulcers	−	Microscopy	4.7	4.5	153	41.4	25.1	Normal	Mycoplasma	1.8 × 10^7^	Cure	
	39	51	M	Iraq	Multiple skin ulcers	+	Microscopy	4.7	4.4	155	36.3	24.1	Normal	Mycoplasma	1.7 × 10^7^	Cure	
	40	34	M	Iraq	Multiple skin ulcers	−	Microscopy	5.9	5.0	160	40.7	24.6	Normal	None	4.4 × 10^7^	Cure	
	41	36	M	Uzbekistan	Single skin ulcers	−	Microscopy	9.3	5.1	352	41.8	36.7	Normal	None	6.0 × 10^6^	Cure	
	42	32	M	Iraq	Multiple skin ulcers	−	Microscopy	4.6	4.7	239	46.3	31	Normal	Legionella	5.0 × 10^7^	Cure	

WBC: white blood cell; RBC: red blood cell; PLT: platelet; ALB: albumin; GLB: globulin; Cure means PCR negative in bone marrow for leishmania detection at the end of treatment; HPS: hemophagocytic syndrome; M: male; F: female; +: positive; −: negative; HBV: hepatitis B virus; un: undetected

### Potential target fragment selection

Out of 34 genes of *Leishmania* with sequence polymorphism previously published, 21 were further analyzed according to the inclusion criteria as follows: first, these gene fragments were shown as markers for the molecular characterization of *Leishmania* strains and species; second, they are common genetic polymorphism sites for the four species (*L. donovani*, *L. infantum*, *L. major* and *L. tropica*); third, gene fragments can obtain from NCBI database among different species and strains (Additional file 1: Table S1).

**Fig. 1:**
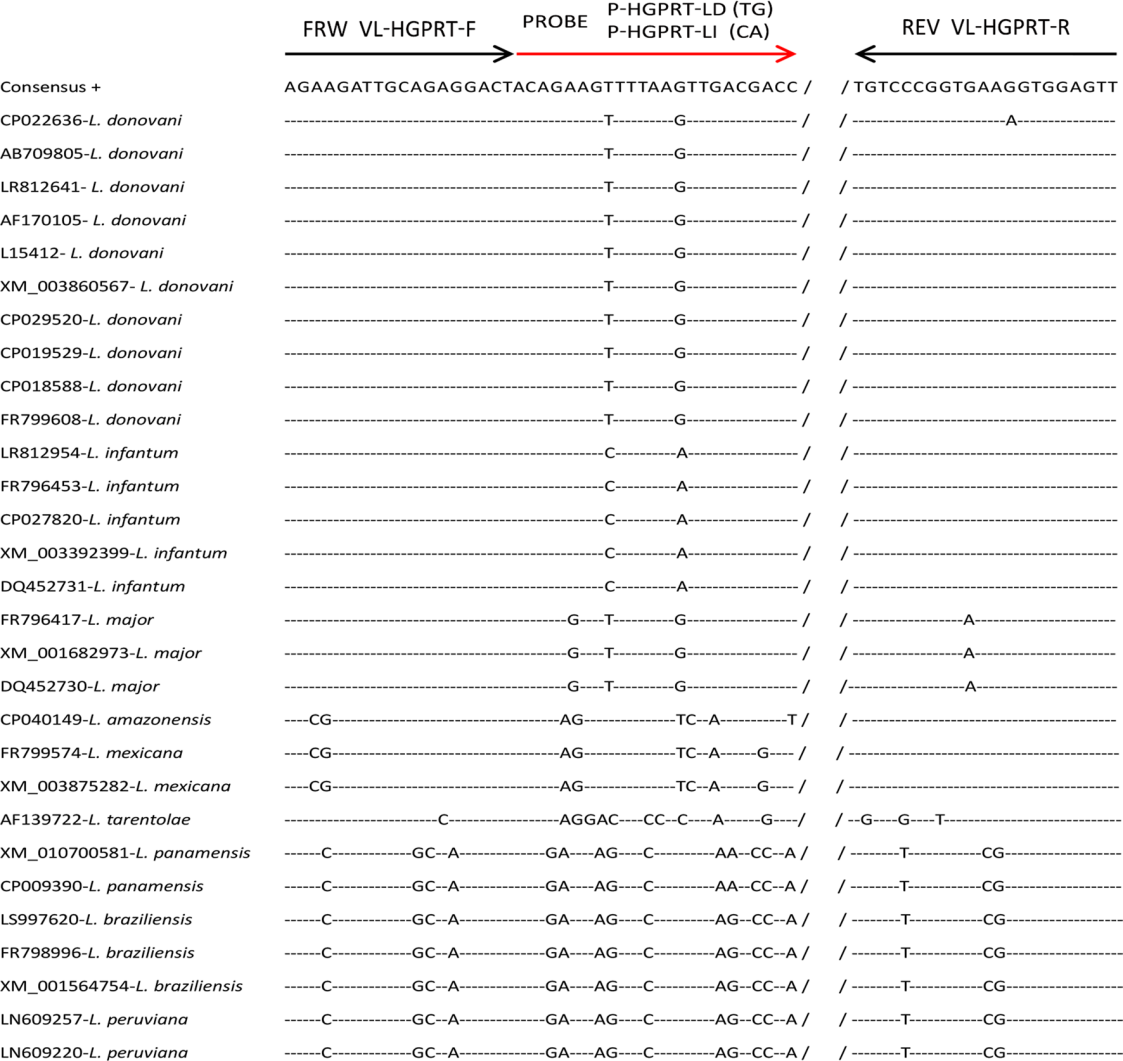
Alignment of HGPRT gene fragment sequences from nine *Leishmania* species with illustrations of primers and probes using for VL species identification between *L. donovani* and *L. infantum*. *HGPRT* Hypoxanthine-guanine phosphoribosyl transferase, *VL* Visceral leishmaniasis

**Fig. 2:**
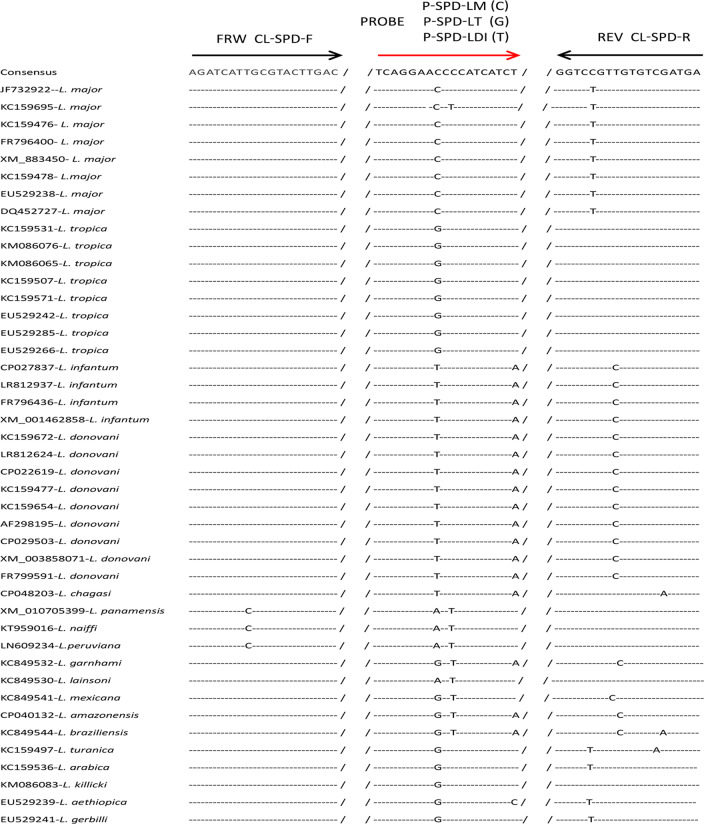
Alignment of SPDSYN sequences from 18 *Leishmania* species with illustrations of primers and probes using for CL species identification among *L. major*, *L. tropica* and *L. donovani/infantum*. *SPDSYN* Spermidine synthase, *CL* Cutaneous leishmaniasis

Each gene sequence among different species of *Leishmania* parasites were analyzed using MLSTest software (v1.0.1.23, institute de Patologia experimental Universidad Nacional de Salta Argentina, Boston, MA, USA), individually, and genes with sequence polymorphisms and species-specific SNPs were screened out (Additional file 2: Table S2). Furthermore, these sites with species-specific SNPs that can be completely distinguished *Leishmania* species which were selected, specifically, the optimal site that can identify species between *L. donovani* and *L. infantum* for VL and distinguishing species among *L. major*, *L. tropica* and *L. donovani*/*L. infantum* for CL were selected as targets (Figs. 1 and 2).

### Primers and probes design and plasmids construction

Twenty-nine HGPRT sequences from nine different species of *Leishmania* parasites and 43 SPDSYN sequences from 18 different *Leishmania* parasites were collected from NCBI database and aligned using BIOEDIT software (v7.0.1, Ibis Biosciences, Carlsbad, CA, USA). Primers were designed based on the conserved region of sequence and probes were designed based on regions with species-specific SNPs of HGPRT genes between *L. donovani* and *L. infantum* and species-specific SNPs of SPDSYN genes among *L. major*, *L. tropica* and *L. donovani/L. infantum* using PRIMER EXPRESS 3.0 (Applied Biosystems-Roche, Branchburg, America) (Table 2)*.* The sequences of the designed primers and probes were tested against the NCBI nucleotide database using the BLASTn (Basic Local Alignment Search Tool) to confirm the species specificity.

**Table 2:** Sequence of primers and probes for the real-time PCR for CL and VL identification

Diseases	Target gene	Species	Primer and probe	Sequences	Amplicon size (bp)	GeneBank accession no.
CL	SPDSYN		CL-SPD-F	5ʹ-AGATCATTGCGTACTTGAC-3ʹ	202	
			CL-SPD-R	5ʹ-TCATCGACACAACAGACC-3ʹ		
		*L. major*	P-SPD-LM	5ʹ-VIC-TCAGGAA**C**CCCATCATCT-MGB-NFQ-3ʹ		KC159695
		*L. tropica*	P-SPD-LT	5ʹ-FAM-TCAGGAA**G**CCCATCATCT- MGB-NFQ-3ʹ		KM086079
		*L. donovani/infantum*	P-SPD-LDI	5ʹ-Texas Red -TCAGGAA**T**CCCATCATCA- MGB-NFQ-3ʹ		AF298195
VL	HGPRT		VL-HGPRT-F2	5ʹ-AGAAGATTGCAGAGGACT-3ʹ	145	
			VL-HGPRT-R1	5ʹ-AACTCCACCTTCACCGGGACA-3ʹ		
		*L. donovani*	P-HGPRT-LD	5ʹ-FAM- ACAGAAG**T**TTTAA**G**TTGACGACC-MGB-NFQ-3ʹ		AB709805
		*L. infantum*	P-HGPRT-LI	5ʹ-VIC- ACAGAAG**C**TTTAA**A**TTGACGACC- MGB-NFQ-3ʹ		XM_003392399

CL: cutaneous leishmaniasis; VL: visceral leishmaniasis; SPDSYN: spermidine synthase; HGPRT: hypoxanthine-guanine phosphoribosyl transferase

### DNA extraction

DNA was extracted from 200 µl bone marrow or 20 mg skin lesion tissue using a DNeasy Blood & Tissue Kit (Qiagen, 69506, Hilden, Germany) according to manufacturer’s instructions and DNA was stored at – 20 ℃.

### Positive control plasmid construction

The HGPRT fragment of *L. donovani* and *L. infantum*, and SPDSYN fragment of *L. major* and *L. donovani/infantum* were amplified from identified clinical specimens and the fragment purified with DNA purification kit (TIANGEN, DP214, Beijing, China). The amplified HGPRT and SPDSYN fragments were ligated into plasmid pUC19 (TAKARA, 3219, Tokyo, Japan) using *Eco*RI and *Hin*d III sites, individually. The correct cloning of the desired target DNA in the recombinant plasmid was confirmed by PCR amplification and DNA sequencing. Due to lack of *L. tropica* parasite and clinical samples from patients with *L. tropica* infection, SPDSYN fragment of *L. tropica* was synthesized based on sequence (Accession no. KM086079) and ligated into plasmid pUC19 by Sangon Biotech Co., Ltd, and then confirmed by PCR amplification and DNA sequencing.

### An allele-specific real-time PCR assay for identification of *Leishmania* parasites

The allele-specific real-time PCR was conducted in a 20 μl reaction volume. For VL species identification, a reaction containing 10 μl of Promega GoTaq Probe qPCR Master Mix (Promega, A6101, Madison, WI, USA), 800 nmol/l forward primer VL-HGP-F2, 800 nmol/L reverse primer VL-HGP-R1, 450 nmol/L hydrolysis Probe P-HGP-LD (5′FAM/3′MGB-NFQ) and P-HGP-LI (5′VIC/3′MGB-NFQ), individually, plus 1 μl template DNA (5–50 ng). While for CL species identification, a reaction containing 10 μl of Promega GoTaq Probe qPCR Master Mix, 300 nmol/L forward primer CL-SPD-F, 300 nmol/L reverse primer CL-SPD-R, 450 nmol/L hydrolysis probes P-SPD-LM (5′VIC/3′MGB-NFQ), P-SPD-LT (5′FAM/3′MGB-NFQ) and P-SPD-LDI (5′Texas Red/3′MGB-NFQ), respectively, plus 1 μl template DNA (5–50 ng). The reaction was performed in the Applied Biosystems 7500 Fast real-time PCR System (ABI) with 95 ℃ for 2 min followed by 40 cycles of 95 ℃ for 15 s, 62 ℃ (VL) and 58 ℃ (CL) for 50 s. Each sample was tested with replicates, the plasmid constructed above were used as positive control and reaction without template DNA (distilled water) was used as negative control in all experiments.

### Analytical sensitivity and specificity of the allele specific real-time PCR for identification of *Leishmania* species

The limit of detection (LOD) of the allele-specific real-time PCR assay was defined as the minimum number of parasites that could be detected based on 8 repeated tests. We used cultured *L. infantum* promastigotes enumerated under a microscope and diluted with blood obtained from healthy volunteer as 1,000, 100, 50, 25, 12, 6, 3 or 1 parasites/μl. Total DNA was extracted from each dilution. The LOD was defined based on the experimentally derived assay precision (intra-assay SD < 0.5 and inter-assay CV < 5%). The specificity of the allele-specific real-time PCR assay was tested with other DNA samples obtained from *P. falciparum*, *T. gondii, B. melitensis* and *O. tsutsugamushi.*

Two plasmids HGPRT/pUC19 of *L. donovani* and *L. infantum* and three plasmids SPDSYN/pUC19 of *L. major*, *L. tropica* and *L. donovani/infantum* were serial dilution as 10^2^, 10^3^, 10^4^, 10^5^, 10^6^, 10^7^, 10^8^, 10^9^ copies/μl, individually. For testing the ability of identification among different species, and the PCR reaction efficiency was evaluated using single template and multiple templates, respectively.

### Evaluation the performances of allele-specific real-time PCR assay for *Leishmania* species identification with clinical samples

Total 42 clinical specimens were tested (Table 1), including 22 bone marrow from VL patients and 20 skin lesions from CL patients. These samples were tested according to the standard procedure described above. The amplification products of 42 clinical samples were sequenced with pair ends by Sangon Biotech Co., Ltd. The results of the new method were compared with sequencing method and the consistence was evaluated.

### Construction the phylogenetic tree using HGPRT and SPDSYN gene fragments to validate the performance of the new assay

Total 51 HGPRT gene sequences were used for phylogenetic tree constructed, including 29 sequences with 9 species obtained from NCBI database and 22 sequences of clinical samples from patients with VL. For construction of CL phylogenetic tree, total 63 SPDSYN gene sequences were used, containing 43 SPDSYN gene sequences with 18 species obtained from NCBI database and 20 sequences of clinical samples from patients with CL. Using MEGA 7.0 software (Mega Limited, Auckland, New Zealand) to build N-J (Neighbor Joining) evolutionary tree based on Kimura 2 algorithm, Statistical support was evaluated by 1,000 bootstrap replications.

## Results

### Targets selection for identification of *Leishmania* species

According to the inclusion criteria described in “Material and methods”, 21 genes were screened out from 34 genes, which were previously reported to exhibit sequence polymorphism among *Leishmania* species (Additional file 1: Table S1). Further analysis indicated that the identity of these 21 genes were 88.3‒99.8% among different species and total 1,970 polymorphism sites were observed within them (Additional file 2: Table S2). Our further bioinformatics analysis were performed to select appropriate SNPs from these 1,970 polymorphism sites for *Leishmania* species identification. The alignment of 29 sequences of HGPRT from nine *Leishmania* species indicated that two SNPs can distinguish between *L. donovani* and *L. infantum* (Fig. 1). Moreover, 1‒2 SNPs were found by comparison of 43 sequences of SPDSYN from 18 *Leishmania* species, which can distinguish *Leishmania* species among *L. major*, *L. tropica* and *L. donovani/L. infantum* well (Fig. 2). Thus, two potential targets for *Leishmania* species identification, HGPRT and SPDSYN, were screened out for further investigations.

### Development of allele specific real-time PCR assay for *Leishmania* species identification

To verify the potential application of HGPRT and SPDSYN in *Leishmania* species identification, the primers and probes were designed according to the conserved sequence of HGPRT and SPDSYN and the SNPs screened out above (Table 2).

**Fig. 3:**
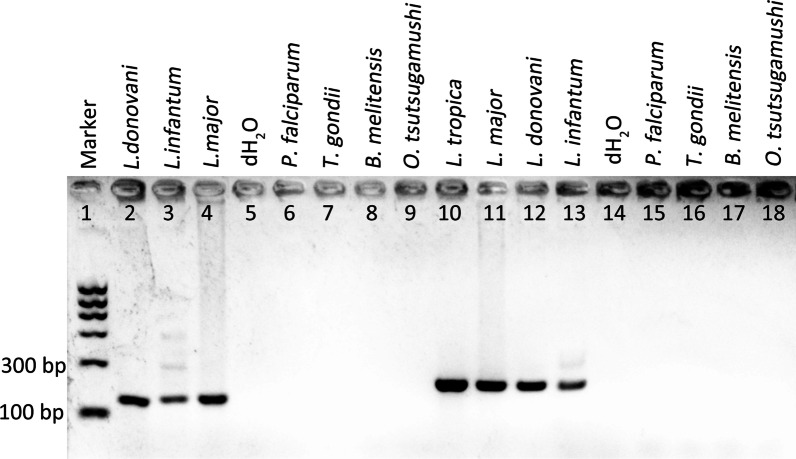
Specificity of the primers designed for HGPRT (lane 2‒9) and SPDSYN (lane 10‒18) amplification. The allele specific real time PCR assays exhibit no cross-reactions with *Plasmodium falciparum*, *Toxoplasma gondii, Brucella melitensis* and *Orientia tsutsugamushi*

Firstly, PCRs were performed with template from clinical samples or constructed plasmids. As expected, the primers, VL-HGPRT-F2 and VL-HGPRT-R1 for HGPRT and CL-SPD-F and CL-SPD-R for SPDSYN, can amplify a 145 bp fragment from *L. donovani*, *L. infantum* and *L. major* samples, and 202 bp fragment from *L. major*, *L. tropica*, *L. donovani* and *L. infantum* samples respectively. In addition, these two pair of primers didn’t recognize any DNA from samples of *P. falciparum*, *T. gondii, B. melitensis and O. tsutsugamushi* (Fig. 3). These results indicated that the targets we selected here were specific for *Leishmania* species detection, which were potentially appropriate for further allele-specific real-time PCR assay construction.

Then an allele specific real-time PCR assay for *Leishmania* species identification were established using the primers and probes described above. Our results showed that this assay can detect 3 parasites/reaction for VL by targeting at HGPRT and 12 parasites/reaction for CL with SPDSYN (Additional file 3: Table S3).

**Fig. 4:**
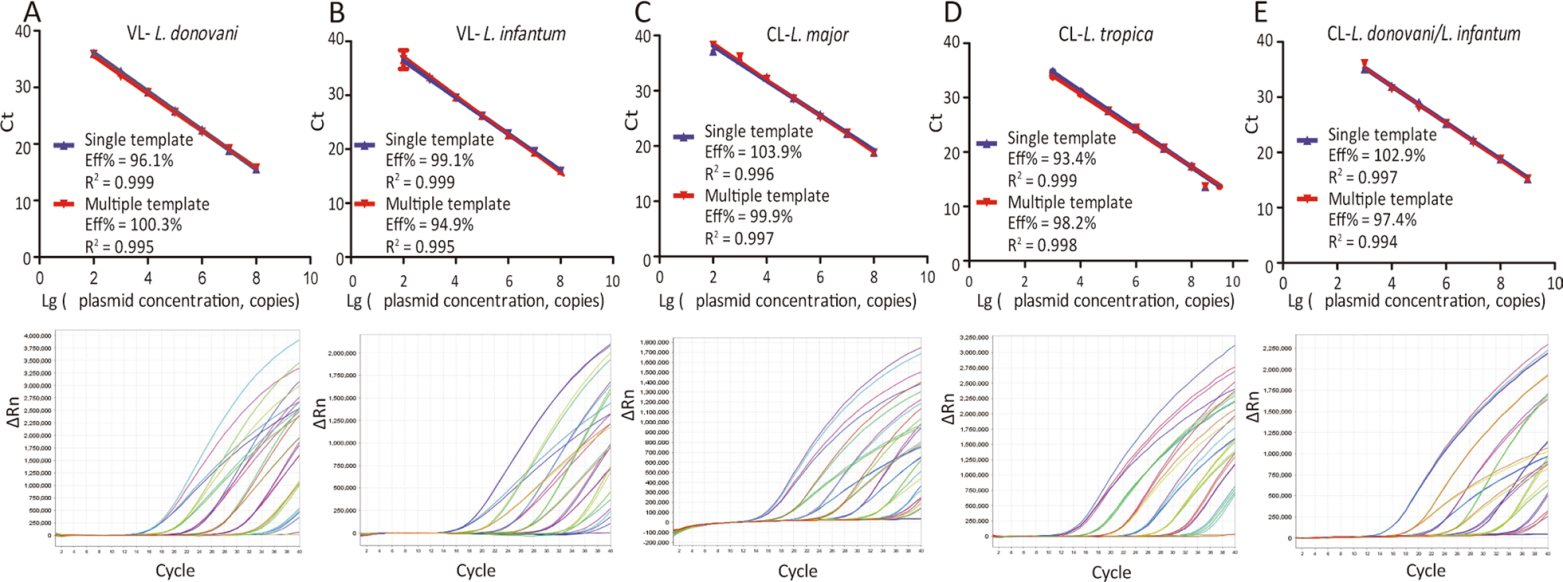
Quantitative correlation between gene copy number and threshold cycle of the allele specific real-time PCR assay. **A**, **B** HGPRT plasmid was serially diluted from 10^2^ to 10^8^ copies/reaction and subjected to allele specific qPCR. Linear regression of Ct vs lg copy number of HGPRT plasmid were generated using single and multi-species templates for *L. donovani* (**A**) and *L. infantum* (**B**) detection, individually. **C** SPDSYN plasmid was serially diluted from 10^2^ to 10^8^ copies/reaction and subjected to allele specific qPCR. Linear regression of Ct vs lg copy number of SPDSYN plasmid were generated using single and multi-species templates for *L. major* detection. **D**, **E** SPDSYN plasmid was serially diluted from 10^3^ to 10^9^ copies/reaction and subjected to allele specific qPCR. Linear regression of Ct vs lg copy number of SPDSYN plasmid were generated using single and multi-species templates for *L. tropica* (**D**) and *L. donovani/infantum* (**E**) detection, respectively. ΔRn = Rn (normalized reporter)-baseline. *Ct* Cycle threshold

The standard curves of this assay were also obtained using serially diluted plasmid DNA. It showed the PCR efficiency with both single-species and multi-species samples reactions were similar and the amplification curve were coincident as well (Fig. 4). The linear were over a 7-log range with a correlation coefficient (R^2^) of 0.995‒0.999 for VL (Fig. 4A and B) and 6/7-log range with a R^2^ of 0.994‒0.999 for CL (Fig. 4C, D and G).

Moreover, both intra-CV% and inter-CV% of Ct values for 20 replicates were < 2% (Additional file 4: Table S4). All these results implied that this allele-specific real-time PCR assay exhibited high precision for VL and CL species identification.

### Validation the established *Leishmania* species identification assay

As the allele-specific real-time PCR assay we developed above exhibited high PCR efficiency and precision, total 42 clinical samples were used to validate the performance of this assay (Table 3). For 22 clinical VL samples, the new method detected 3 as *L. donovani* infections and 19 as *L. infantum*, which was consistence with the sequencing results. Similarly, 20 skin lesion CL samples were all identified as *L. major* using by this new method and confirmed by sequencing as well.

**Table 3:** The allele-specific real-time PCR results with the samples from 42 patients

Diseases	Patients ID	Testing results Ct (SD)		Sequencing results	Diseases	Patients ID	Testing results Ct (SD)			Sequencing results
		*L. donovani*	*L. infantum*				*L. major*	*L. tropica*	*L. donovani /infantum*	
VL	1	un	30.7 (0.3)	*L. infantum*	CL	23	30.1 (0.3)	un	un	*L. major*
	2	un	34.4 (0.1)	*L. infantum*		24	30.0 (0.3)	un	un	*L. major*
	3	un	34.8 (0.3)	*L. infantum*		25	26.4 (0.1)	un	un	*L. major*
	4	un	33.7 (0.2)	*L. infantum*		26	28.2 (0.2)	un	un	*L. major*
	5	un	33.2 (0.2)	*L. infantum*		27	29.7 (0.2)	un	un	*L. major*
	6	un	36.8 (0.4)	*L. infantum*		28	32.3 (0.6)	un	un	*L. major*
	7	un	27.1 (0.1)	*L. infantum*		29	35.6 (0.4)	un	un	*L. major*
	8	un	31.0 (0.1)	*L. infantum*		30	26.2 (0.1)	un	un	*L. major*
	9	un	33.8 (0.2)	*L. infantum*		31	25.6 (0.1)	un	un	*L. major*
	10	29.43 (0.16)	un	*L. donovani*		32	27.2 (0.2)	un	un	*L. major*
	11	27.66 (0.15)	un	*L. donovani*		33	32.3 (0.4)	un	un	*L. major*
	12	un	27.0 (0.3)	*L. infantum*		34	29.4 (0.2)	un	un	*L. major*
	13	un	35.9 (0.4)	*L. infantum*		35	27.6 (0.2)	un	un	*L. major*
	14	un	31.7 (0.3)	*L. infantum*		36	28.3 (0.4)	un	un	*L. major*
	15	un	36.1 (0.5)	*L. infantum*		37	28.3 (0.6)	un	un	*L. major*
	16	un	38.5 (0.4)	*L. infantum*		38	29.8 (0.1)	un	un	*L. major*
	17	un	33.4 (0.7)	*L. infantum*		39	28.8 (0.2)	un	un	*L. major*
	18	un	26.4 (0.1)	*L. infantum*		40	26.4 (0.4)	un	un	*L. major*
	19	34.45 (0.39)	un	*L. donovani*		41	29.1 (0.4)	un	un	*L. major*
	20	un	31.2 (0.1)	*L. infantum*		42	35.0 (0.4)	un	un	*L. major*
	21	un	29.2 (0.3)	*L. infantum*						
	22	un	25.3 (0.3)	*L. infantum*						

*Ct* cycle threshold, *SD* standard deviation, *VL* visceral leishmaniasis, *CL* cutaneous leishmaniasis, *un* undetected

**Fig. 5:**
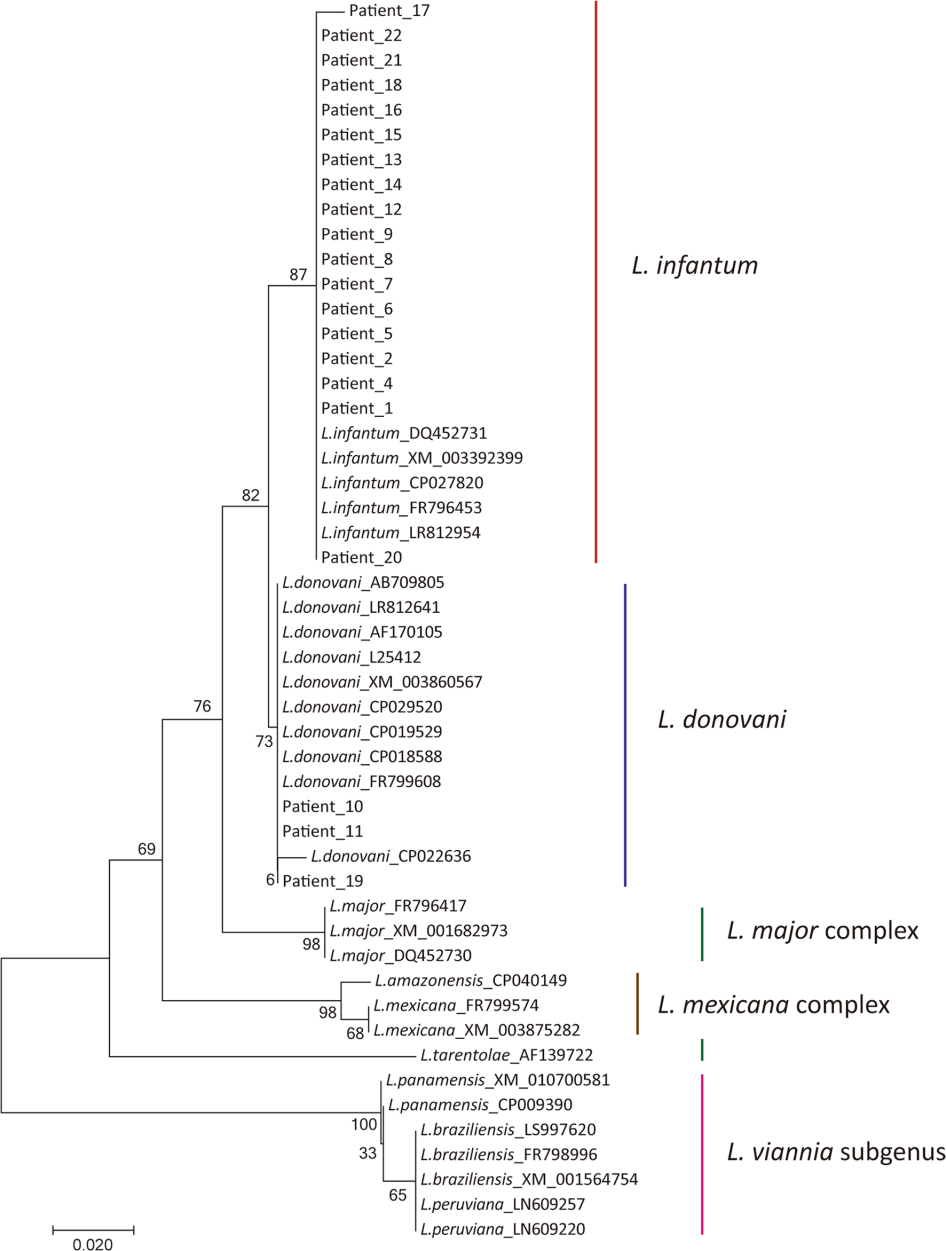
Neighbor-Joining was used to generate phylogenetic tree with 1000 replications for bootstrap based on HGPRT gene fragment, including sequences from patients’ samples

**Fig. 6:**
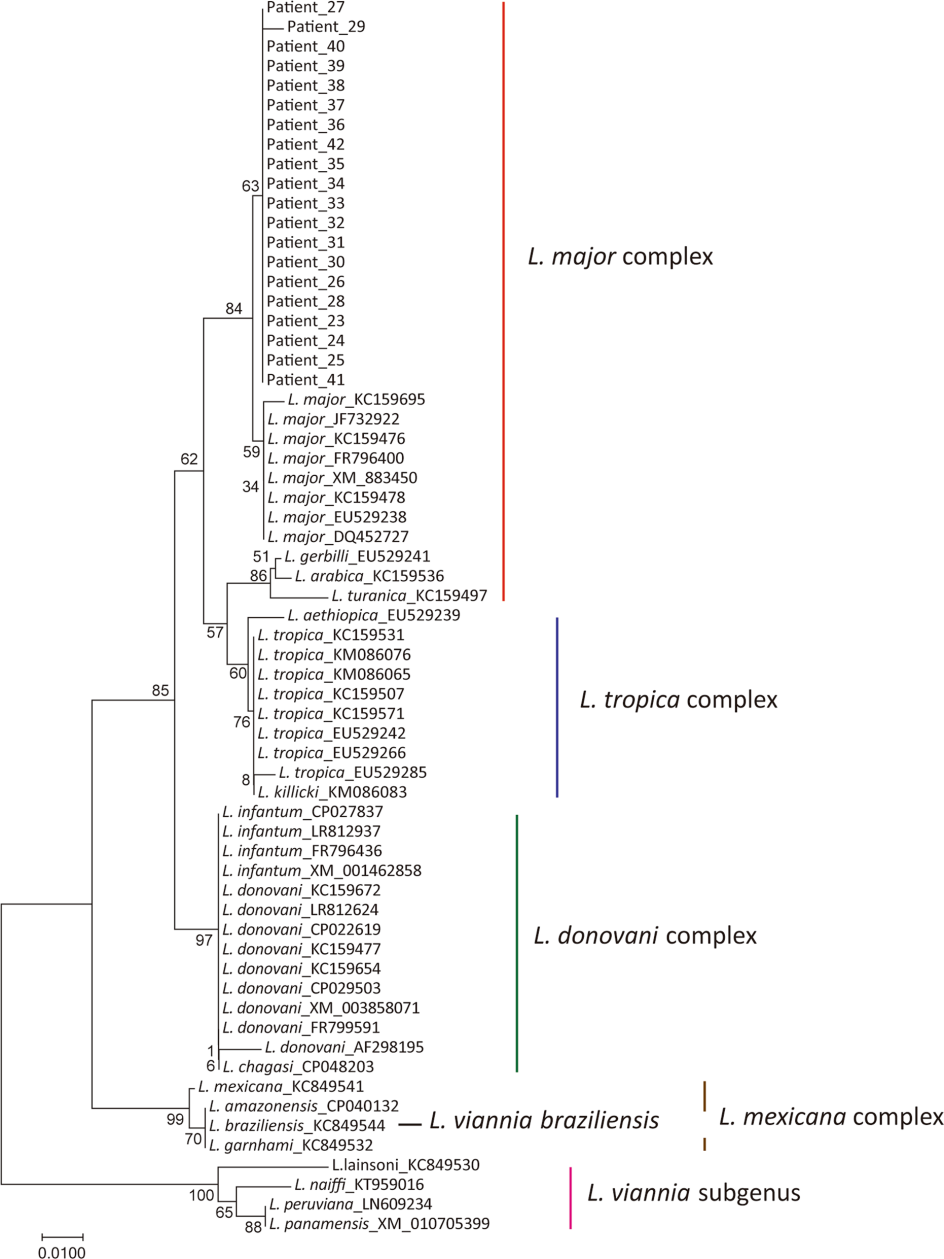
Neighbor-Joining was used to generate phylogenetic tree with 1000 replications for bootstrap based on SPDSYN gene fragment, including sequences from patients’ samples

A phylogenetic tree was constructed using 29 *Leishmania* HGPRT sequences (145 bp) from nine *Leishmania* species and 22 VL clinical samples. The clustering results shows that 3/22 clinical samples (patient ID 10, 11, 19) were clustered with *L. donovani* and 19/22 clinical samples were clustered with *L. infantum* (Fig. 5 and Table 3). Also, phylogenetic analysis with 43 SPDSYN gene sequences (202 bp) from18 *Leishmania* species and 20 CL clinical samples indicated that 20 clinical samples were all clustered with *L. major* (Fig. 6 and Table 3). Both of these two clustering outcomes were consistence with the new methods we developed here, which further confirmed the reliability of this new assay for *Leishmania* species identification.

## Discussion

In this study, HGPRT and SPDSYN genes, which exhibit species-specific SNPs, were selected based on the screening of 21 housekeeping gene sequences from 9 species of VL and 18 species of CL. According to the conserved regions and species-specific SNPs, primers and probes were designed to perform two allele specific real-time PCR assays respectively. Our results showed that this new developed assay could identify the *Leishmania* species for VL between *L. donovani* and *L. infantum* with HGPRT and for CL among *L. major*, *L. tropica* and *L. donovani*/*L. infantum* with SPDSYN.

Previous studies identified *Leishmania* species using a SYBR-green based qPCR followed by melting analysis. Several different target were in these assays, including ITS1 for *Leishmania* (*Viannia*) spp., *L. donovani* complex, *L. tropica*, *L. mexicana*, *L. amazonensis*, *L. major*, and *L. aethiopica* [33]; canine beta-2-microglobulin and human glyceraldehyde-3 phosphate dehydrogenase for *Leishmania* (*Viannia*) spp*.*, *L. infantum* and *L. amazonensis* [34]; amino acid permease 3 and cytochrome oxidase II (COII) genes for *L. major*, *L. tropica* and mix infections [35]; minicircle kDNA for the subgenera *Leishmania* and *Viannia* [36]; Cyt b gene for *L. braziliensis*, *L. guyanensis*, *L. infantum*, *L. major*, *L. tropica* and *L. panamensis* [27], and glucose-6-phosphate dehydrogenase for *L. braziliensis* or *L. peruviania* from the other *Leishmania* (*Viannia*) spp [32]. Although this type of assay was simple and cost-consuming, it is less specific and the results analysis was more complicated compared to the probe-based real-time PCR [25, 37].

There are also some real-time PCR identification methods were developed with different detecting targets, such as cathepsin L-like cysteine protease B gene for *L. major*, *L. tropica* and *L. aethiopica* [38]; amino acid permease 3 (AAP3) and COII for *L. major* and *L. tropica* [39], and glucose phosphate isomerase (GPI) for *Leishmania* (*Viannia*) spp*.*, *L. mexicana* complex, *L. infantum*/*donovani* complex and *L. major* complex [31]. The two allele-specific qPCR assays we developed here were focused on the *Leishmania* species that are common in clinical practice, such as *L. donovani*, *L. infantum*, *L. major* and *L. tropica*. Using two firstly reported targets, HGPRT and SPDSYN genes with species-specific SNPs, the LOD of these assays was 3 and 12 parasites/reaction for VL and CL, individually and no cross-reaction with *P. falciparum*, *T. gondii*, *B. melitensis*, *O. tsutsugamushi* and non-target species of *Leishmania* was detected (Additional file 3: Table S3; Figs. 4 and 5). Considering it takes only 2.5 h to identify *Leishmania* species directly from clinical samples without parasites isolation or culture, these assays are suitable in clinical practice.

A total of 42 clinical samples (22 VL and 20 CL) were used to evaluate the performance of the allele-specific real-time PCR assay, which identified 22 VL clinical samples as *L. donovani* (*n* = 3) and *L. infantum* (*n* = 19), 20 CL clinical samples as *L. major* (*n* = 20). These results were consistent with the following sequencing analysis, which indicated that these new tools can distinguish SNPs among different *Leishmania* species well (Table 3). Further phylogenetic analysis was performed to validate the results of these allele-specific qPCR assays, which confirmed their reliability for potential clinical applications (Figs. 5 and 6).

HGPRT gene encoded hypoxanthine phosphoribosyl transferase, which is a central enzyme in the purine recycling pathway of all protozoan parasites [40]. Spermidine synthase encoded by SPDSYN gene is a key enzyme in the polyamine biosynthetic pathway of protozoan parasites [41]. These two housekeeping gene sequences exhibit observed interspecies polymorphism, which imply that our assays in this study could be applied to distinguish not only *Leishmania* species we described here, but also other species not included in this study. Indeed, our phylogenetic analysis implied that the sequence of HGPRT gene could differentiate more *Leishmania* species than we tested here, including *L. major*, *L. mexicana* complex and *Leishmania* (*Viannia*) subgenus (Fig. 5). Meanwhile, SPDSYN gene fragment appears to be able to distinguish *Leishmania* (*Viannia*) *braziliensis*, *L. mexicana* complex and *Leishmania* (*Viannia*) subgenus as well (Fig. 6). Further investigations are worthwhile to be performed to extend the potential scope of these identification assays.

Broad variations are noted in efficacies of leishmaniasis treatment depending on the *Leishmania* species, which identification would be helpful in clinical practice. For example, antimonial and miltefosine are more effective to *L. major* and *L. donovani* than *L. infantum* [16, 42, 43]. Unlike *L. major*, *L. tropica* appears unresponsive to PM-based ointments [44, 45]. Amphotericin B is used to treat *L. tropica* or *L. major* related CL, but not *L. infantum* [46–48]. The efficacy rates of azoles for *L. infantum*, *L. donovani*, *L. major* and *L. tropica* were 88%, 80%, 53% and 15%, respectively [49]. Further, *Leishmania* species-specific administrations were applied for better clinical efficiency. For *L. tropica* infection, intralesional treatment was more efficient than intramuscular administration with sodium stibogluconate [50]. Intravenous antimonial treatment could produce better cure rates against *L. panamensis* or *L. braziliensis* related CL compared with *L. major* [51–53]. Thus, as a rapid and accurate tool for *Leishmania* species identification, it would be helpful for species-adapted therapeutic schedule and patient management.

Unfortunately, MLEE, the “gold standard” method for *Leishmania* species identification, could not be performed in this study, due to only a few of parasites can be cultured in vitro from our stored clinical samples. Instead of MLEE, phylogenetic analysis of HGPRT and SPDSYN was applied to further confirm our species distinguish results. Although our new methods with HGPRT and SPDSYN can distinguish between *L. donovani* and *L. infantum* of VL and among *L. major*, *L. tropica* and *L. donovani*/*infantum* of CL accurately, a larger sample size should be investigated in future for further confidence, especially with clinical samples of *L. tropica* infection and different species co-infection which were not applied in this study.

## Conclusions

A novel probe-based allele-specific real-time PCR assay was established with newly reported targets, HGPRT and SPDSYN, which could identify *Leishmania* species between *L. donovani* and *L. infantum* for VL, and among *L. major*, *L. tropica* and *L. donovani*/*infantum* for CL. This method could be applied for not only *Leishmania* species-adapted therapeutic management but also ecological and epidemiological studies.

## Supplementary Information

**Additional file 1: Table S1.** 34 housekeeping genes of *Leishmania* with sequence polymorphism.

**Additional file 2: Table S2.** *Leishmania* interspecies polymorphism in 21 genes.

**Additional file 3: Table S3.** The sensitivity of allele-specific real-time PCR assay.

**Additional file 4: Table S4.** Precision of intra and inter-assay of allele-specific real-time PCR assay.

## Data Availability

All data generated or analysed during this study are included in this published article.

## Abbreviations

VL: Visceral leishmaniasis
CL: Cutaneous leishmaniasis
ML: Mucosal leishmaniasis
PKDL: Post kala-azar dermal leishmaniasis
RFLP: Restriction fragment length polymorphism
MLEE: Multi-site enzyme electrophoresis
HGPRT: Hypoxanthine-guanine phosphoribosyl transferase
SPDSYN: Spermidine synthase
SNPs: Single nucleotide polymorphisms
LOD: Limit of detection
AAP3: Amino acid permease 3
COII: Cytochrome oxidase II
GPI: Glucosephosphate isomerase
PM: Paromomycin
